# Neurovirulent Vaccine-Derived Polioviruses in Sewage from Highly Immune Populations

**DOI:** 10.1371/journal.pone.0000069

**Published:** 2006-12-20

**Authors:** Lester M. Shulman, Yossi Manor, Danit Sofer, Rachel Handsher, Tiberio Swartz, Francis Delpeyroux, Ella Mendelson

**Affiliations:** 1 Central Virology Laboratory, Public Health Services, Ministry of Health, Chaim Sheba Medical Center Tel Hashomer, Israel; 2 Israel Center for Disease Control, Ministry of Health, Chaim Sheba Medical Center Tel Hashomer, Israel; 3 Unit of Molecular Prevention and Therapy of Human Diseases, Centre National de la Recherche Scientifique FRE 2849, Pasteur Institute Paris, France; 4 Faculty of Life Sciences, Bar Ilan University Ramat Gan, Israel; National Institute for Communicable Diseases, South Africa

## Abstract

**Background:**

Vaccine-derived polioviruses (VDPVs) have caused poliomyelitis outbreaks in communities with sub-optimal vaccination. Israeli environmental surveillance of sewage from populations with high (>95%) documented vaccine coverage of confirmed efficacy identified two separate evolutionary clusters of VDPVs: Group 1 (1998–2005, one system, population 1.6×10^6^) and Group 2 (2006, 2 systems, populations 0.7×10^6^ and 5×10^4^).

**Principal Findings:**

Molecular analyses support evolution of nine Group 1 VDPVs along five different lineages, starting from a common ancestral type 2 vaccine-derived Sabin-2/Sabin-1 recombinant strain, and independent evolution of three Group 2 VDPVs along one lineage starting from a different recombinant strain. The primary evidence for two independent origins was based on comparison of unique recombination fingerprints, the number and distribution of identical substitutions, and evolutionary rates. Geometric mean titers of neutralizing antibodies against Group 1 VDPVs were significantly lower than against vaccine strains in all age-group cohorts tested. All individuals had neutralizing titers >1∶8 against these VDPVs except 7% of the 20–50 year cohort. Group 1 VDPVs were highly neurovirulent in a transgenic mouse model. Intermediate levels of protective immunity against Group 2 VDPVs correlated with fewer (5.0+1.0) amino acid substitutions in neutralizing antigenic sites than in Group 1 VDPV's (12.1±1.5).

**Significance:**

VDPVs that revert from live oral attenuated vaccines and reacquire characteristics of wild-type polioviruses not only threaten populations with poor immune coverage, but are also a potential source for re-introduction of poliomyelitis into highly immune populations through older individuals with waning immunity. The presence of two independently evolved groups of VDPVs in Israel and the growing number of reports of environmental VDPV elsewhere make it imperative to determine the global frequency of environmental VDPV. Our study underscores the importance of the environmental surveillance and the need to reconsider the global strategies for polio eradication and the proposed cessation of vaccination.

## Introduction

Enhanced inactivated polio vaccine (eIPV) is an enhanced potency injectable trivalent vaccine produced from wild-type poliovirus strains representing each of the three poliovirus serotypes [1]. The polioviruses in eIPV have been inactivated with formalin. In contrast, OPV is a trivalent attenuated live virus vaccine where the poliovirus of each serotype was derived by selecting progeny of wild poliovirus that had lost their neurovirulence. Between 1990 and 2005, infants in Israel received three doses of eIPV and three doses of OPV poliovirus vaccines by the age of 15 months [2], followed by an OPV booster in first grade (age five to six). After 2005, the routine immunization schedule was changed and consisted of 4 eIPV by 15 months and an eIPV booster in first grade. From 1999, national vaccination coverage ranged between 92% and 95% and within individual health districts from 81 to 100%. Annual sero-surveys documented >95% immunity to all three strains consistent with previous reports [2]. The Palestinian Health Authority and/or UNRWA administered a similar combined OPV/eIPV vaccination program to all children in the West Bank and Gaza districts supplemented with bi-annual National Immunization Days for children 0 to 5 years old. As a consequence both Israel and the Palestinian territories have been free of poliomyelitis in the last decade [3], [4].

All three poliovirus serotypes have high mutation rates [5], [6]. Polioviruses of all three serotypes in Sabin live tri-valent OPV, like all other polioviruses, readily accumulate mutations during passage in human hosts. Nucleotide misincorporation into the viral genome occurs at a fairly constant rate [3], [7]. This rate can be used as a rough “molecular clock” to estimate evolutionary intervals between isolates [3], [8]. Some vaccine progeny revert to wild phenotype (as only 2 to 3 nucleotide substitutions differentiate between the two phenotypes) [9] and this reversion to neurovirulence may even occur during infection of the primary vaccinee [10]. Circulating polioviruses also frequently recombine with genomes of other polio and non-polio enteroviruses in primary or subsequent hosts [11]–[14]. Recombination junctions generally clustered in particular subgenomic regions that were dependent on the serotype of the isolate and/or on the associations of genomic segments in recombinants[15]. However, since these preferential recombination sites are not very precise hot spots, it is broadly accepted that shared recombinations imply common ancestry.

A poliovirus isolate is classified as vaccine, vaccine-derived (VDPV), or wild type poliovirus based on the percent nucleotide sequence homology between its capsid protein VP1 and that of the corresponding OPV vaccine serotype. VP1 homology of 99–100% is classified as vaccine virus, 85% to 99% as VDPV and <85% as wild type poliovirus [16]. VDPVs evolve from vaccine along one of two known pathways indicated by the letter “i” or “c” preceding the word “VDPV”. VDPVs that arise during persistent infection of immunodeficient individuals are termed iVDPV [17]. VDPVs that evolve during continuous transmissions of vaccine virus between unvaccinated individuals in populations with low vaccination coverage [17]–[19] or when vaccination programs are interrupted and sufficient numbers of unimmunized infants accumulate [20] are termed “circulating VDPV” (cVDPV).

In recent years vaccine-derived poliovirus (cVDPVs) have caused poliomyelitis outbreaks in developing countries with low immunization coverage [18]. In general, unvaccinated sub-populations are protected by herd immunity when vaccination rates are sufficiently high [21]. However, there are exceptions, the most recent being circulation of VDPV in the Amish community in Minnesota, USA [22].

The aim of monthly surveillance of sewage from sentinel communities initiated in Israel in 1989 is to monitor circulation of poliovirus in the absence of poliomyelitis, to characterize the polioviruses that circulate and to asses the risk for reemergence of poliomyelitis [4], [23]. The sensitivity of environmental surveillance depends on many factors such as population size, sample frequency and physical properties of the sewage system [24]. Each sewage isolate was characterized as OPV, VDPV, or wild type by serological and molecular methods [16]. Extensive molecular analysis of each VDPV isolate is a requirement to determine whether it arose as a cVDPV or iVDPV. Distinction between circulating virus and virus excreted by one or at most a few individuals, is obviously imperative for assessing the risk posed by such isolates.

In this report we characterize 12 highly diverged VDPVs isolated between 1998 and 2006 from two geographically separated Israeli population with a high documented immune profiles and address possible risk of re-emergence of disease. One or more individuals may have continuously excreted these highly neurovirulent VDPVs into the environment during this interval. Molecular analyses of these VDPVs indicate that the isolates could be divided into two groups that evolved independently. Isolates within each group shared a common evolutionary origin from an ancestral Sabin2/Sabin1 recombinant strain. In one group 9 isolates diverging along 5 separate lineages while in the other 3 isolates diverged within a single lineage. These findings raise serious concern about reemergence of VDPVs into the circulating pool of neurovirulent polioviruses.

## Results

### Presence of 12 VDPVs in sewage between 1998 and 2006

Poliovirus presence in sewage systems in Israel and the Palestinian territories has been monitored monthly in sentinel sites since 1989. These sewage systems include one in central Israel in the Tel Aviv area which serves a population of 1.6 million and another serving 0.7 million out of the total population in Jerusalem and surrounding communities (described in more detail in the electronic supplement). In the 17 years between 1989 and 2006, there were no VDPVs found at any sentinel sites in Israel, the Gaza District and the West Bank other than those reported in this manuscript that were isolated from the Tel Aviv area in central Israel between 1998 and 2006 and from the Jerusalem area in 2006. VDPV negative sentinel sites included Haifa, Beersheba, Rahat, Gaza district and various communities in the West Bank.

On 6 occasions between 1998 and 2004, sewage collected at the entrance to the main treatment plant of the central Israeli sewage system in the Tel Aviv area (Primary Site #1; Figure S1) contained polioviruses with characteristics that differed from OPV. These viruses grew at 40°C, were not neutralized by Sabin 1, 2, or 3 specific monoclonal antibodies and their RNA did not hybridize with vaccine specific probes in dot blot hybridization analyses [25]. For reference, >50 samples were collected from this sentinel site prior to the 1998 and none contained VDPVs.

After the 6^th^ isolate was obtained in 2004, 5 additional monthly sampling sites were chosen at the mouths of major tributaries within the system to increase the chance of isolating more VDPVs and to more precisely locate the site of their entry into the sewage system (Secondary Sites #1-A to #1-E; Figure S1). In 2005, 3 additional VDPV isolates were obtained from sewage collected at one of these secondary sites. The specific site was located at the mouth of a trunk line serving a population of 800.000 (Site #1-A, Figure S1). As a consequence, this branch of the sewage system was subdivided into sub-regions monitored at 3 new tertiary sites (Tertiary Sites (#1-A-1 to 1-A-3, Figure S1). In 2006 two VDPVs were isolated from one of these tertiary sites (Tertiary Site #1-A-1). Interestingly, while this site (Site 1-A-1) represents a small population averaging 50,000 individuals, a large part of this population is constantly turning over since the major contributors of sewage to this subdivision are the hotels along the Mediterranean seashore.

One month prior to isolation of the 2 VDPVs from the tertiary site in the central region of Israel, a single VDPV was isolated from a totally unrelated sewage system. The sample containing this isolates was a pooled sewage sample representing a major portion (approximately 700,000 individuals) of the population of Jerusalem and the surrounding municipalities (Site #2, Figure S1). Between 1998 when VDPV was first found in Israel in the Tel Aviv area and the end of 2005, >105 samples were analyzed from the Jerusalem area covered by the pooled sample and all were negative for VDPVs.

The quality of sampling and sample processing was uniformly high. For example, polio vaccine was isolated from 62% of sewage samples collected between 1998 and 2002 at the primary site in Tel Aviv (Site #1). Following introduction of enterovirus monitoring in 2002, enterovirus was isolated from >97% of the samples from this primary site, and from >97% of the samples from secondary and tertiary sites. Similarly, polo vaccine was isolated from 64% of all individual samples collected between 1998 and 2002 within the Jerusalem areas represented by the pooled VDPV positive sample and enterovirus was isolated from 96% of the samples collected from 2002 to the end of 2005.

Genomic sequences of the 12 VDPVs have EMBL access numbers, AJ288062, AM040035-39, AM056049-50, AM158275-6, and AM292219-21. Access numbers for Sabin 1 and Sabin 2 sequences are V01150 and X00595, respectively. The EMBL alignment access number for the first 9 VDPVs is DS 63108. The names of the isolates as filed in the EMBL database have been simplified for this manuscript (see Table S1). They have been re-named SDs to indicate that they were Sabine derived. In addition all SD isolates have a two digit numbers indicating the year of isolation. In cases where more than 1 VDPV was isolated within a given year, an additional digit indicates the order of isolation (i.e. isolate PV2-5104-1_ISR99 (AM04003X) being the third type 2, VDPV isolate in Israel in 1999 has been renamed SD-99-3).

### Evidence for a shared evolutionary pathway for 9 VDPVs isolated between 1998 and 2005

**Figure 1:**
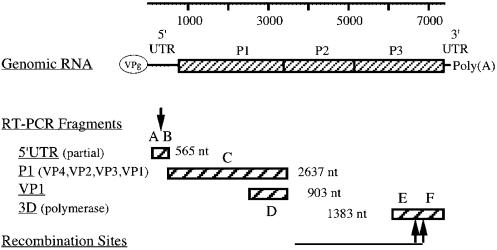
Genomic fragments of type 2 VDPVs amplified by RT-PCR for sequencing and phylogenetic analysis. The polyprotein encoded by the Sabin 2 genome is represented by a rectangle on the genomic RNA. The 5′ UTR (partial), P1, VP1, and 3D polymerase regions that were amplified by RT-PCR and sequenced are represented by 4 rectangles with dark cross hatching below the genomic RNA. They correspond to nt 183 to 747, 748 to 3384, 2482 to 3384, and 5986 to 7368, respectively, of Sabin 2 (Acc: X00595). The downward pointing arrow indicates the start of the hypervariable region of 5′UTR. The upward-pointing arrow indicates the location of the genetic recombination in the 3D polymerase gene. The letters R1 through R6 correspond to the separate regions used for sequence comparisons and phylogenetic analysis as shown in Figs. 2, 3 and 4.

The entire genomes of the first 5 VDPV isolates [26] were sequenced. For the 4 subsequent VDPVs partial genomic sequences were obtained that included a major portion of the 5′UTR, the complete P1 region encoding all 4 structural proteins (capsid proteins VP1, VP2, VP3, and VP4), and the non-structural viral polymerase, 3D. A diagram of the genomic regions that were analyzed is shown in Fig. 1. Since the VP1 sequence of all 9 isolates diverged from Sabin-2 by 8.7%–14.0% [Table S2] and their source and evolutionary path were unknown, these VDPV isolates will hereinafter be referred to as ambiguous aVDPVs [17].

A non-vaccine origin had previously been ruled out for the first aVDPV, SD-98 [8], because its VP1 sequence was genetically much closer to Sabin 2 than to any of the wild type polioviruses found in the Middle East or elsewhere since 1980. The VP1 regions in all 8 subsequent VDPV isolates also showed the highest degree of homology with Sabin 2 (not shown).

**Figure 2:**
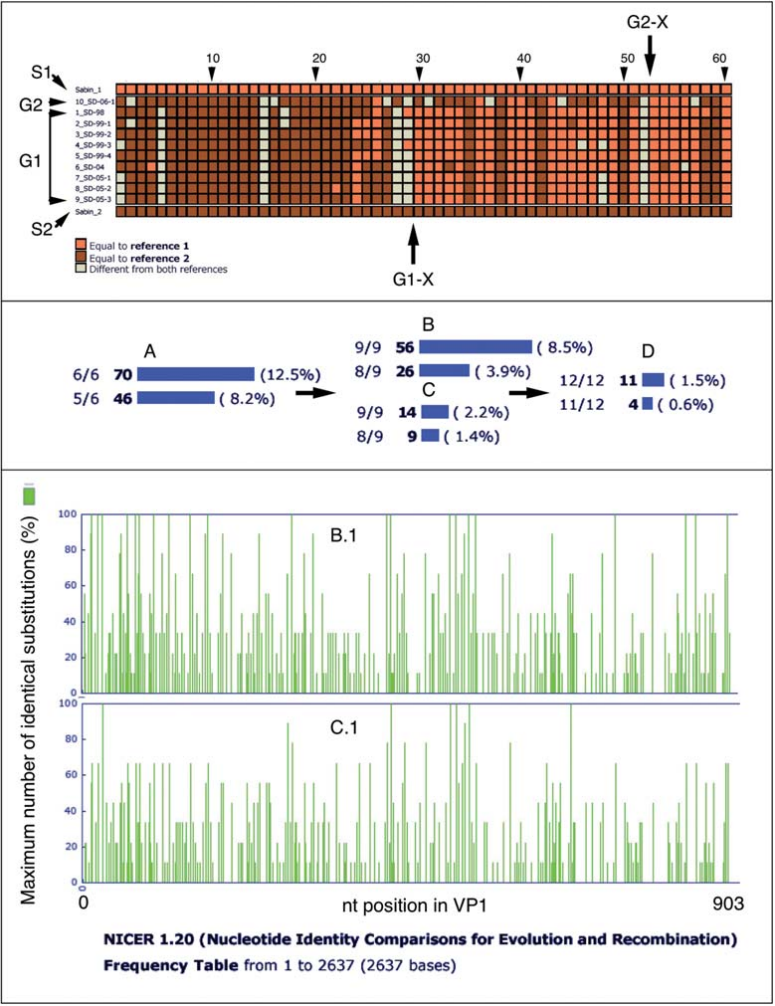
NICER Identity Analysis of the aVDPVs: genomic recombination fingerprint in the 3D polymerase gene, and identity plots for the P1 and VP1 regions. **Upper panel:** An Identity Plot created by the NICER program of the nucleotide sequences of Group 1 **(G1)** and Group 2 **(G2)** aVDPVs compared with Sabin 1 (S1) and Sabin 2 **(S2)** at each nucleotide position of 3D where Sabin 1 and Sabin 2 differ. The nucleotides of the query sequences are scored as identical to Sabin 1 **(light orange/light grey(B&W))**, identical to Sabin 2 **(dark brown/dark grey(B&W))**, or different from both **(grey/white(B&W))**. The upward pointing arrow **(G1-X)**, indicating the most likely position where identity of the Group 1 sequences shifts from Sabin 2 to Sabin 1, has been placed between positions 19 and 20 which corresponds to nucleotides 6693 and 6705 of Sabin 2 (Acc: X00595). The downward pointing arrow **(G2-X)**; indicating the most likely position where identity of the Group 2 sequences shifts from Sabin 2 to Sabin 1, has been placed between positions 52 and 53 which corresponds to nucleotides 6771 and 6774 of Sabin 2 (Acc: X00595). **Middle panel:** An Identity Frequency plot to indicate the frequency of occurrence of nucleotide positions in P1 (region R3) of the reference sequence (Sabin 2) where the equivalent nucleotide on at least one of the query sequences differs was created by the NICER program. The number and frequency of identical positions (all of query sequences differ and all have the identical substitution; N/N) and near identical positions (all but one of the query sequences have identical substitutions; N-1/N) is shown for analysis of 6 Group 1 aVDPVs isolated between 1998 and 2004 **(A)**; the 6 Group 1 isolates in A plus the 3 Group 1 aVDPVs isolated in 2005 **(B)**; the 6 Group 1 isolates in A plus the 3 Group 2 aVDPVs isolated in 2006 **(C)**; and all 9 Group 1 and 3 Group 2 isolates **(D)** is shown. For example; in plot A there were 70 positions where 6 of 6 query sequences had identical substitutions and this identity occurred in 12.5% of all positions where at least one of the query sequences differed from the reference sequence. **Bottom panel:** An Identity Frequency Distribution plot to indicate the nucleotide position where the equivalent nucleotide on at least one of the query sequences differs from the nucleotide in VP1 (region R4) of the reference sequence (Sabin 2) and % of query sequences that had the maximum number of identical substitutions at each of those positions. Analysis of the 6 Group 1 aVDPV isolates plus either the 3 Group 1 aVDPVs isolated in 2005 **(B.1)** or the 3 Group 2 aVDPVs isolated in 2006 **(C.1)**.

Determining the degree to which a group of poliovirus isolates share common features such as genomic recombinations and identical substitutions allows assessment of their relatedness. An important finding in the evolutionary development of these 9 VDPVs is that they all share the same genomic recombination that occurred in the middle of the 3D polymerase gene at positions equivalent to nucleotides 6693 and 6705 of Sabin 2 (Acc: X00959). Interestingly, at this point homology shifts from Sabin 2 to Sabin 1. This common recombination point is shown in an Identity Plot generated by the NICER program (upper panel of Fig. 2). This common recombination can be confirmed by similarity analyses using programs such as SimPlot (see Figure S2). This common recombination fingerprint provides strong evidence that all 9 aVDPVs evolved from a common ancestral Sabin2/Sabin1 recombinant.

Further evidence that all 9 aVDPVs were highly related comes from the large numbers of identical substitutions observed in all 9 aVDPV sequences when comparing them to Sabin 2. Non-coding regions and coding regions of structural and non-structural genes may be subjected to different evolutionary pressures. Therefore each of these regions was subdivided into 2 regions that were analyzed separately (Fig. 1). The two segments of the 5′ untranslated region (5′UTR) that were analyzed were a highly conserved 450 nucleotide region (R1), and a hypervariable 67 nucleotide region [27] (R2). The next two analyzed segments consisted of 2637 nucleotides of the entire structural genomic region, P1 (R3) and 903 nucleotides of capsid protein VP1 (R4). The final two segments encoding non-structural genes included a 669 nucleotide region of the viral RNA polymerase 3D gene located before the site of recombination (R5) and a 705 nucleotide region of 3D located after the recombination site (R6).

In regions R1 through R6, Identity Analysis using the NICER program revealed 13, 10, 56, 21, 25 and 18 nucleotide positions, respectively, that had identical nucleotide substitutions relative to Sabin 2 in all 9 aVDPVs. In addition, there were 5, 2, 26, 8, 14, and 4 nucleotide positions in regions R1 through R6, respectively, where 8 of the 9 VDPVs had identical substitutions. The Identity Analysis results for region R3 can be seen in the graph labeled “B” in the middle panel of Fig. 2 and the results for region R4 can be seen in the graph “B.1” in the bottom panel of Fig. 2. Graph “B” shows the frequency of identical (9 of 9) and near identical (8 of 9) substitutions. Graph “B.1” indicates each nucleotide position along the viral genome where at least 1 of the 9 aVDPV different from Sabin 2 and the per cent of all isolates with highest number of identical substitutions at that position.

Despite a maximum inter-isolate divergence of 15.4% [Table S2], these molecular data strongly suggest that all 9 aVDPVs evolved from a common ancestral type2 vaccine-derived Sabin2/Sabin1 recombinant strain. As recognition of this shared evolutionary origin, these 9 aVDPVs will be referred to as Group 1 aVDPVs.

### Phylogenetic analysis

**Figure 3:**
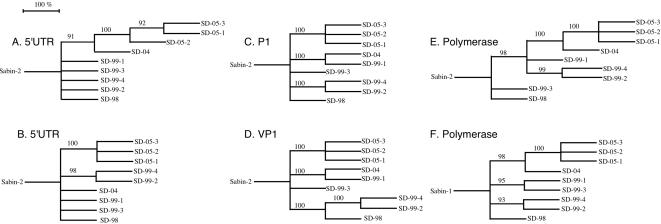
Phylogenetic relationships among Type 2 aVDPVs at 6 locations along the genome. Phylogenetic trees 3A through 3F are maximum likelihood phylogenetic trees for 6 regions distributed along the polioviral genome, two in the 5′ non-coding region, 2 in the region encoding structural genes and 2 in the region encoding non-structural genes (R1–R6 in Fig. 1). The non-coding regions analyzed are **R1**, the 5′UTR encoding domains III to VI (Fig. **3A**; nt 196 to nt 645), and **R2**, the 5′UTR hypervariable region (Fig. **3B**: nt 646 to nt 712). The regions encoding structural genes analyzed are **R3**, the P1 region encoding all capsid proteins (Fig. **3C**; nt 748 to nt 3384), and **R4**, VP1 (Fig. **3D**; nt 2482 to nt 3384). The regions encoding nonstructural genes are **R5**, the RNA polymerase gene before the recombination point (Fig. **3E**; nt 5986 to nt 6690), and **R6**, the RNA polymerase gene after the recombination point (Fig. **3F**; nt 6700 to nt 7368). Nucleotide positions are given relative to Sabin 2 (Acc: X00595). Trees were prepared using the dnaml maximum likelihood application of the PHYLIP program. The transition:transversion ratio was set at 10, data was bootstrapped 100 times and the order of sequences for each bootstrap randomized 3 times. Trees A through E are rooted to Sabin 2, while tree F is rooted to Sabin 1(Acc: V01150). Branch lengths correspond to the number of times a given branch appears among all bootstrapped trees. All branches with bootstrap values <90% have been collapsed. The trees were visualized using the njplot program.

Evolutionary relatedness of the 9 Group 1 aVDPVs has also been assessed by construction of phylogenetic trees. Separate maximum likelihood phylogenetic trees were prepared for regions R1 through R6 (Figs. 3A–F, respectively). Data was bootstrapped 100 times and only branches that appeared in >90% of the trees formed from the 100 bootstrapped data sets were depicted. The trees shown are true rooted trees since the ancestral sequences are known, i.e. Sabin 2 for regions R1 to R5, and Sabin 1 for region R6. The 9 aVDPVs group into 5 major lineages for VP1 (region R4, Fig. 3D). Interestingly the topologies of the trees from the other genomic regions were very similar but not completely super-imposable (note alterations in branch positions of SD-99-2, SD-99-4 and SD-04). Similarity in the topology of genes derived from Sabin 1 and genes derived from Sabin 2 strongly suggests that the recombination occurred before most of the other mutations, probably soon after vaccination. Such intratypic recombinations in vaccine recipients are common [10].

### Estimating the time of the initiating exposure

Three different methods used to estimate the time of initial exposure to Sabin 2 of Group 1 aVDPVs yielded 3 different estimations for the date of initial exposure to Sabin 2: 1991–1996; 1986 and 1985–1987. All three approaches were based on rates of nucleotide substitutions in capsid protein VP1. The first two methods analyzed only synonymous codon substitution rates while the third method included the overall rate of synonymous and non-synonymous substitutions. Synonymous codon substitutions do not alter the amino acid composition of the viral protein, whereas, non-synonymous codons might subject progeny to negative or positive selection. Thus the rate of appearance of synonymous codons probably more closely represents the true rate of nucleotide substitutions. We have used the common analytical approach (based on synonymous codons alone) that looks at substitutions in the 3^rd^ nucleotide position where most synonymous substitutions are found. It also excludes double substitutions since the order of substitutions is unknown making it almost impossible to determine whether the change at the 3^rd^ position was synonymous or not.

Our first approach to evaluating the time of initial exposure assumed that the aVDPV evolved at the same rate as other polioviruses (3% 3^rd^ position synonymous substitutions per year) [3], [8], [17], [28]. A simple division of the % of synonymous substitutions for each isolate by 3% indicated that initial exposure to Sabin 2 occurred sometime between 1991 and 1996. This broad range questions the basic assumption that the annual substitution rate was 3%.

**Figure 4:**
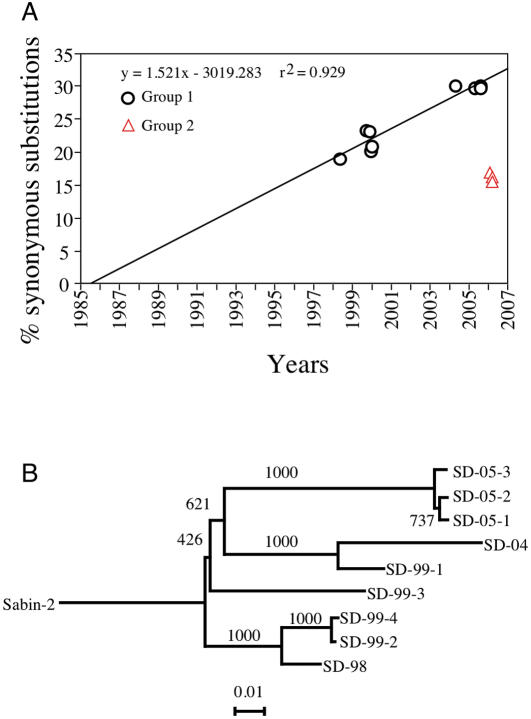
Estimation of the date of initial exposure to Sabin-2. **Fig. 4.A.** is a method for estimating of the date of initial exposure based on regression analysis of the accumulation of the % of 3^rd^ position synonymous substitutions from all codons with single nucleotide substitutions in VP1 (region R4 in Fig. 3D). The date of initial exposure to Sabin 2 was determined by extrapolation backwards to 0% substitutions. (r^2^ = 0.886). Group 1 isolates are represented by open circles while Group 2 Isolates are represented by open triangles. **Fig. 4.B** is a rooted, neighbor-joining, phylogenetic tree of the same region, region, R4, analyzed in Figs. 3D and 4.A. Unlike Fig. 4.A, substitutions in all three codon positions were taken into account. The tree was prepared with ClustalX. Data was bootstrapped 1000 times. The numbers on the branches are the bootstrap values. The tree was visualized using the njplot program. Branch lengths correspond to genetic distances. Since dates of isolation are know, branch lengths were converted to time. The time for the distance between Sabin 2 and the first branch was calculated proportionally to the minimum and maximum branch “times” between different pairs of aVDPVs.

A more rigorous approach was to extrapolate, a linear regression of the % accumulation of 3^rd^ position synonymous substitutions versus the date of isolation back to 0%. These results (Fig. 4A) imply that initial exposure occurred in 1986 and the rate of misincorporation was much slower than the reported rate for other polioviruses [3], [8], [17], [28].

The third approach involved construction of a bootstrapped, neighbor joining phylogenetic tree rooted to Sabin 2 (Fig. 4B). Branches of such a tree are proportional to evolutionary distance, and conversely the entire tree is proportional to the time of evolution along individual branches. Since the time between isolation of each aVDPV is known, the time of evolution along each of the different branches can be determined. The proportional time range for the entire tree was estimated by determining the ratio between the length of the branches with the minimum and maximum times of evolution and the length of the entire tree. The results indicate that first exposure occurred between 1985–1987 and support the slower rate of evolution indicated by the regression analysis.

### Evidence that the 3 aVDPVs isolated in 2006 are related but evolved independently from Group 1 aVDPVs

The 3 aVDPVs isolated in 2006 were subjected to the same types of analysis as the Group 1 aVDPVs. Like Group 1 aVDPVs, they had evolved from Sabin 2. The VP1 and P1 regions of the aVDPV from Jerusalem were very closely related to those of the two isolates from the central Tel Aviv region (Table S2). A simple neighbor joining tree rooted to Sabin 2 indicated that they belonged to a 6^th^ lineage (not shown). However after more intensive analysis assisted by the NICER program, evidence accumulated suggesting that these 3 aVDPVs had evolved independently from the previous 9, Group 1 aVDPVs and thus belong to a new evolutionary group which we will call Group 2.

Firstly, Group 2 isolates recombined with Sabin 1 in the polymerase gene, however most significantly at a point different from those of all Group 1 aVDPVs (compare arrow G1-X between nucleotide positions 6693 and 6705 with arrow G2-X between nucleotide positions 6771 and 6777 in the upper panel, Fig. 2.).

Secondly, these Group 2 isolates did not share most of the identical (9/9) and near identical (8/9) substitutions shared by the 9, Group 1 aVDPVs. This is illustrated by the sequential comparative identity analysis results (NICER analysis) shown in the middle and lower panels of Fig. 2. For the middle panel, the P1 genomic regions (region R3) of the 6 aVDPVs isolated between 1998 and 2004 were compared with Sabin 2. The number of identical (6/6) and near identical (5/6) substitutions, 70 and 46,, respectively were used a baseline for determining whether subsequent aVDPV isolates evolved from the same Sabin 2 progenitor or from a different Sabin 2 progenitor. In the examples shown, identical (9/9) and near identical (8/9) substitutions were recalculated for these 6 and either the 3 related Group 1 aVDPVs from the single lineage in 2005 or the three related Group 2 aVDPVs from the single lineage in 2006 (B and C, respectively, middle panel, Fig. 2). The number of identical and near identical substitutions decreased slightly to 56 and 26, respectively, when the remaining 3, Group 1 aVDPVs were added (Graph B). In contrast, they dropped precipitously to 14 and 9, respectively when the 3 Group aVDPV isolates were included (Graph C) and even lower when all 12 aVDPVs were analyzed (Graph D). The bottom panel shows the distribution along the genome within VP1 (region R4) of all nucleotide positions where at least one of the isolates differed from Sabin 2 and the % maximum number of identical substitutions at that location. The combinations of isolates analyzed for Graphs B.1 and C.1 were identical to those for Graphs B and C, respectively. The local and global distribution of identical substitutions is clearly qualitatively very different when results for related isolates (6 plus 3 Group 1 aVDPVs) are compared with results for the 6 Group 1 and 3 Group 2 aVDPVs.

Thirdly, the rate of accumulation of nucleotide substitutions of the 3 Group 2 aVDPVs is not consistent with that for the Group 1 aVDPVs if it is hypothesized that all 12 aVDPVs share a common evolutionary path from the same progenitor Sabin 2 isolate. This is most clearly demonstrated in the regression analysis for determining the common date of initial exposure for the 9 Group 1 isolates (Fig. 4A) by the failure of the Group 2 isolates to fall even close to the Group 1 regression line.

### Characterization of genetic changes with potential phenotypic significance

Only two nucleotide substitutions are responsible for the phenotypic shift from the wild type-2 neurovirulent poliovirus parental strain to the Sabin-2 attenuated vaccine strain [9]. Both of these neurovirulence attenuation sites genotypically reverted to wild type in all 9 Group 1 and 2 aVDPV isolates. Specifically, nucleotide 481 in the 5′UTR reverted from A to G and the ATT codon (isoleucine at position 143) in VP1 was changed into ACT in 8 Group 1 and 3 Group 2 aVDPV or to ACA in one Group 1 aVDPV (both ACT and ACA encode threonine).

Other sites of significance were found in viral genomic regions encoding capsid proteins VP1, VP2 and VP3. These sites encode amino acid sequences that are important for immune recognition. Disease prevention is determined to a large extent by the degree in which neutralizing antibodies against these epitopes in vaccine strains recognize non-vaccine poliovirus. Differences in the amino acid composition at these neutralizing antigenic sites can affect this recognition. The positions of these epitopes for type 2 poliovirus were based on analogy with known sites for types 1 and 3 polioviruses [8], [28]. Computer generated translation of the genes encoding these capsid proteins for the 9 Group 1 aVDPVs indicated 12.1±1.5 amino acid substitutions (range 10 to 15 per aVDPV, Table 1.) within and bordering these neutralizing antigenic sites in all three capsid proteins. In contrast there were 5.0±1.0 (range 4 to 6) substitutions for Group 2 aVDPVs. This clear difference further substantiates division of the isolates into two independent groups.

**Figure 5:**
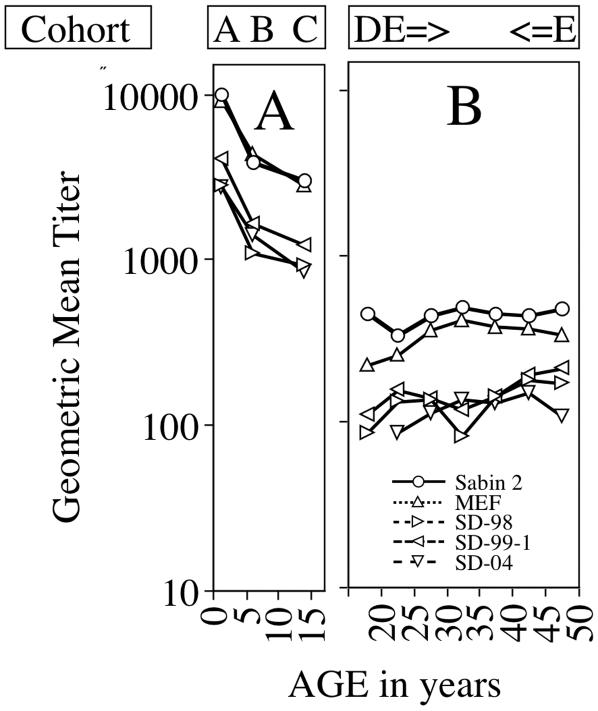
Geometric mean titers (GMT) of neutralizing antibodies against Type 2 strains by age group. The GMT for each strain was calculated for each age group after data was normalized by comparison to titers from a standard pool of 20 positive sera included in each analysis as described in Materials and Methods. The cohort (see Table 2) for which each GMT point was determined is shown at the top of the figure. The symbols for each isolate are in Fig. 5B. **Fig. 5.A** represents GMT of neutralizing antibodies to poliovirus in sera from cohorts aged 15 months to 15 years in which the complete immunization history of all individuals was documented. **Fig. 5.B** represents the GMT in sera from cohorts aged 18 years to 50 years from convenient samples obtained from the serum banks of the Israel Center for Disease Control (ICDC).

### Phenotypic reversion to neurovirulence of aVDPV isolates

The effect of the nucleotide substitutions at both neurovirulence attenuation sites was confirmed in a mouse model. Transgenic mice expressing the human receptor for poliovirus were challenged with 1.0−1.8×10^8^ PFU of Sabin 2, MEF (wild vaccine type 2), S139C6 (a minimally diverged, highly neurovirulent type 1 vaccine strain), and aVDPV isolates SD-98, SD-99-1, SD-99-2 and SD-99-4. The Mean Health Time of 14±0.0 symptom free days [8], [29] for mice challenged with the Sabin 2 vaccine control, was reduced by MEF and S139C6 viruses to 9.6±1.5 and 2.8±0.1 days, respectively. The number of symptom free days after challenge with 1.0×10^8^ PFU of SD-98, SD-99-1, SD-99-2 and SD-99-4 were 3.1±0.1, 7.1±1.6, 3.6±0.2 and 3.1±0.1, respectively. Increasing the challenge dose of SD-98 to 2.8×10^8^ PFU reduced the number of symptom free days to 2.6±0.3. Thus all Group 1 aVDPVs tested were extremely neurovirulent in the animal model.

### Assessment of age-related immunity to aVDPV isolates

To determine whether predicted amino acid substitutions in neutralizing antigenic sites altered the aVDPVs antigenically, thereby affecting the population immunity against them, we compared antibody titers from standard neutralization assays. Four aVDPV isolates, SD-98, SD-99-1, SD-04, and SD-05-1, one from each year and representing 4 of the 5 Group 1 lineages, were tested in parallel with Sabin-2 and MEF using sera obtained from cohorts of different ages (described in Table 2). A more limited survey was carried out comparing the titers against MEF and representative Group 1 (SD-98) and Group 2 (SD-06-1 and SD-06-2) aVDPVs in cohorts F, G, and H (Table 2).

**Table 2:**
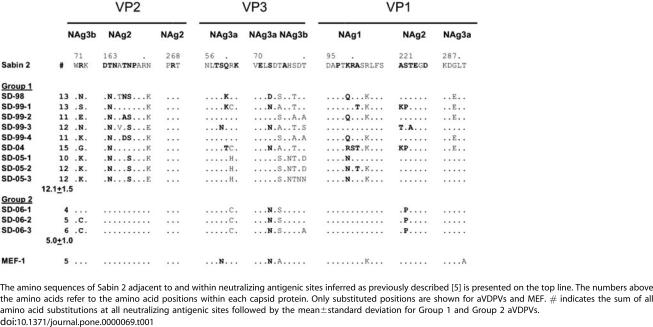
Age-grouped cohorts used for determining GMT to type 2 poliovirus strains.

Cohort	Age in years	Date collected^a^	Number of Samples	Vaccination status
A	1.25	2000; SS	28	Intercalated 3×eIPV plus 3×OPV
B	6	1998; SS	22	Intercalated 3×eIPV plus 3×OPV; OPV booster at 6 yrs
C	14	2004; SS	20	Intercalated 3×eIPV plus 3×OPV; OPV booster at 6 yrs
D	18	1997; ICDC	78	Status unknown; convenient sample
E	21–50	2003; ICDC	150 (5 per year)	Status unknown; convenient sample
F	20–24	2005; ICDC	15 (3 per year)	Status unknown; convenient sample
G	11–12	2003; SS	12	Intercalated 3×eIPV plus 3×OPV; OPV booster at 6 yrs
H	20–30	2004: ICDC	12	Status unknown; convenient sample

a Year serum collected; **SS** (Standard sero-survey; vaccine status available for all members of cohort) or **ICDC** (convenient samples are random samples from the Israel Center for Disease Control serum bank)

b The basic intercalated vaccination program in Israel between 1990 and 2005 consisted of a dose of enhanced inactivated polio vaccine (eIPV) at 2 months, combined administration of eIPV and oral polio vaccine (OPV) at 4 months, OPV at 6 months, and concurrent administration of eIPV and OPV between 12 to 15 months.

The first step was to evaluate the titers of neutralizing antibodies against the vaccine strains in age-grouped cohorts A through E (Table 2). The geometric mean titers (GMT) for antibodies neutralizing Sabin-2 and MEF, calculated as described [30], declined >10 fold as the age of the cohorts increased from 15 months to 18 years and then stabilized for the next 35 years (p<0.05, Wilcoxon signed rank test for unpaired data) (Fig. 5). The GMT for adults was still protective and the age-related decrease was consistent with previous studies [30].

**Table 1:**
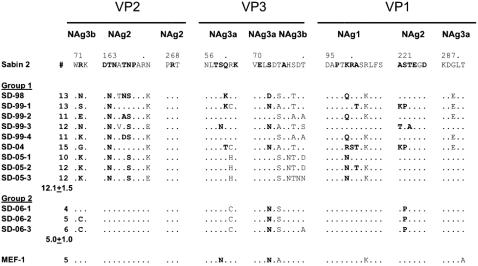
Amino acid substitutions in neutralizing antigenic sites of Israeli aVDPVs.

The amino sequences of Sabin 2 adjacent to and within neutralizing antigenic sites inferred as previously described [5] is presented on the top line. The numbers above the amino acids refer to the amino acid positions within each capsid protein. Only substituted positions are shown for aVDPVs and MEF. # indicates the sum of all amino acid substitutions at all neutralizing antigenic sites followed by the mean±standard deviation for Group 1 and Group 2 aVDPVs.

10.1371/journal.pone.0000069.t001

The next step was to compare the GMT against vaccine strains and representative aVDPVs. At each age group, the GMT against Group 1 aVDPV strains SD-98, SD-99-1 and SD-04 was significantly lower than the GMT of antibodies to vaccine strains (Fig. 5., p<0.005, Wilcoxon signed rank test for paired data). While at some ages, the GMT for one VDPV was significantly different from the others (p<0.05), there was no aVDPV that was consistently different at all time points. Moreover, within the 20–50 year old cohort there were 6 individuals who had neutralizing antibody titers below 1:8 against SD-98, 3 against SD-04 and 1 against both SD-99 and SD-04. In contrast, these 10 individuals were still protected against both vaccine strains. This group had GMTs against Sabin 2 and MEF of 69 and 53, respectively, and all individuals had protective titers (i.e. above 1:8). Thus 10/150 (7%) individuals >21 years old who had protective neutralizing antibody titers against Sabin 2 and MEF had titers below the minimum protective level of 1:8 for at least one or more of the VDPV strains. The GMT against Group 1 isolate SD-05-1 was significantly lower than that against MEF (Table 3) in cohort F.

**Table 3:**
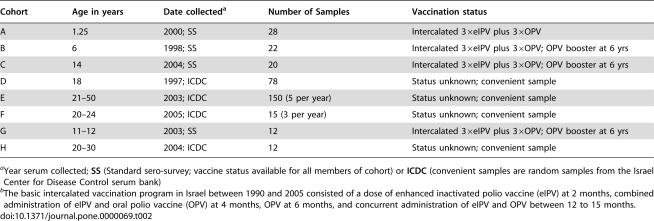
Geometric mean titers (GMT) of neutralizing antibodies against Type 2 strains by age group.

COHORT	MEF-2	SD-98	SD-05-1	SD-06-1	SD-06-2
F	268	n.d.	61*	n.d.	n.d.
G	512	143*	n.d.	406	323*
H	90	50*	n.d.	114	68

* p<0.05 n.d. = not done

GMT against MEF, Group 1 isolate SD-98, and Group 2 aVDPVs SD-06-1 and SD-06-02, were tested in cohorts G and H. Consistent with previous findings, the GMTs for Group 1 SD-98 were significantly lower (p<0.05) then against MEF (Table 3). The GMTs for Group 2 isolates fell between those for MEF and SD-98, however with the exception of SD-06-1 in cohort G, they were not significantly different from MEF.

## Discussion

The efficacy of the Israeli vaccination program has been documented, showing that neutralizing antibody titers for all age groups were >1∶8 against all three OPV and IPV serotypes in >95% of the population [2], [30]. Protective titers drop >10 fold by age 18. The population benefits from this high immunity and has not endured any poliomyelitis outbreaks since 1989 despite numerous wild poliovirus importations into the Gaza district [4], [23]. These incursions were detected in the absence of paralytic disease by environmental surveillance of sewage samples. The environmental surveillance also detected 9 related and highly diverged VDPVs that have been isolated from sewage over the 8-year period, 1998–2005. Six were from a site located at the entrance of the main sewage treatment facility serving 1.6 million people in central Israel where no VDPV were detected in >50 monthly samplings before 1998 or >15 after 2004. Another three VDPVs were isolated from a secondary site located upstream on a trunk line serving 800,000 people. An additional 3 highly diverged VDPV were isolated in 2006, 1 from a separate geographical location, the Jerusalem area, and 2 from one of the trunk lines leading into the secondary site where the 3 isolates were obtained in 2005. All isolates were clearly VDPV since molecular evidence showed closer homology to Sabin 2 than to any wild type 2 polioviruses anywhere since 1980 [8].

It is difficult to determine the number of infected individuals from sewage [24] and the one or more excretors of these VDPVs remain unidentified. The evolutionary path of these isolates is unknown for which reason they have been designated ambiguous aVDPVs [17]. The lack of detection in most 1998–2005 samples probably relates to several factors: the limits of detection [24], the intermittent presence of the excretor(s) in the surveillance area, and/or the percent of stool samples containing poliovirus from known iVDPV sources [28].

A unique recombination marker and a high number of identical nucleotide substitutions indicate that all 9 aVDPVs isolated between 1998 and 2005 evolved from a common Sabin2/Sabin1 recombinant ancestral strain despite ≤15% inter-isolate divergence. This high divergence (Supplement Table 1) and the short time between isolations appear to rule out direct sequential evolution and favor parallel development along at least 5 separate lineages. Divergence commenced well before the first isolates were identified. However, the divergence of the closely related isolates in two lineages appears to have occurred more recently, e.g., SD-99-2 and SD-99–4 in one lineage and the three 2005 isolates in another. For this discussion we designate these 9 aVDPV as Group 1 aVDPVs to indicate that they evolved in parallel, concurrently from a common ancestor. The 3 aVDPVs isolated in 2006 differ from Group 1 isolates by their different recombination pattern, low number of shared identical recombinations, and different apparent date for commencement of evolution, as well as by a different pattern of amino acid substitutions in neutralizing antigenic sites. For this discussion we designate these 3 aVDPVs as Group 2 aVDPVs to indicate that they evolved in parallel, and concurrently but from a different ancestral Sabin 2 isolate than Group 1 aVDPVs.

High sequence diversity by evolution from a common ancestor implies a long chain of replication in one or more human hosts. Each of three events had to have occurred in a single individual although not necessarily the same one: initial exposure to trivalent OPV, intertypic recombination between Sabin 2 and Sabin1 in the polymerase gene and the identical nucleotide substitutions throughout the genome.

Characterization of the early stages of VDPV emergence [11], [31], [32] and the late stages in evolution of iVDPV and cVDPV (Reviewed in [17], [18]) have provided the following guidelines for distinguishing between evolution in a single individual and evolution during a long chain of host-to-host transmission. iVDPVs are characterized by co-evolution of many lineages in a single immune compromised host. They presumably colonize different areas of the gut [28], are excreted intermittently, have numerous amino acid substitutions in neutralizing antigenic sites and if recombine, intratypically recombine with polioviruses [28] but not with clade C enteroviruses. In contrast, cVDPVs are transmitted from one naive host to another and usually have few distinct lineages and significantly fewer amino acid substitutions in antigenic sites. cVDPVs readily recombine with polio and non-polio enteroviruses, probably as soon as they start circulating [32]. The Israeli Group 1, poliovirus type 2 aVDPVs were characterized by multiple lineages, numerous amino acid substitutions in neutralizing antigenic sites, a single intratypic polymerase gene recombination with type1 poliovaccine, no recombinations with clade C non-polio enteroviruses and intermittent isolation patterns. These characteristics are more consistent with iVDPVs. Phylogenetic trees were prepared from different genomic regions of isolates representing different lineages from a single immune-deficient patient[28]. Minor incongruencies of tree branching were used to suggest intratypic recombination between those iVDPV isolates. Recombination can only occur when both isolates replicate within the same infected cell in a single individual. Thus similar incongruence in branching patterns observed between regions for Israeli aVDPVs might also indicate intratypic recombinations between lineages in a single host. If so, this would indicate simultaneous presence in a single host and strengthen the likelihood that isolates are iVDPV. The apparent time interval between initiation of evolution of the aVDPVs and the Sabin 2/Sabin 1 recombination event might be explained by re-exposure of a chronically infected infant with trivalent OPV at age 5 to 6 or by contact with immunized siblings. The location at which the initial trivalent OPV exposure occurred for Group 1 and Group 2 is unknown and may or may not have occurred in Israel. Both Group 1 and Group 2 aVDPVs could have circulated in sub-populations with low vaccine coverage, although such pockets have not yet been identified (see example [33]). Additionally, even highly immunized healthy children can potentially support poliovirus circulation: more than 60% of ≤15 month old infants immunized with 3 IPV and 3 OPV doses and with an excellent humoral immune profile still excreted high poliovirus titers some for up to 3 weeks following re-challenge with OPV [T. Swartz, et al. unpublished].

This novel isolation of several highly related aVDPVs over long time intervals suggests that our advanced surveillance techniques can repeatedly detect iVDPV in a community of 1.6 million individuals. Furthermore, if these Group 1 isolates are iVDPV they represent a significantly longer chronic infection in the absence of poliomyelitis and have a much higher genetic divergence of co-infecting lineages than reported for all identified patients, but one [17]. Furthermore the sequential isolation of the Group 2 aVDPVs in 2 separate geographical locations indicate that when a unified widespread surveillance program has been consistently used, virus circulation or movement of infected individuals between different large populations can be followed.

Recently introduced environmental surveillance programs in South Africa [34] Estonia[35], Slovakia [36], Japan [31] and Russia [37] also yielded aVDPVs. Thus we propose that similar long-term studies should be undertaken worldwide to assess how frequently VDPVs are present in the environment and the potential global risks posed by aVDPVs and iVDPVs. Such assessments are essential before discontinuation of vaccination as proposed in the Global Poliomyelitis Eradication Initiative.

In our study, neutralizing antibody titers against the Group 1 aVDPVs were ≥3.3 fold lower compared to vaccine strains [Fig. 5]. These titers dropped below protective levels in 7% of immunized adults. Accumulation of adults with waned immunity may be the equivalent of accumulation of naïve infants when polio vaccination is interrupted[20]. Significantly, poliomyelitis occurred in individuals above the age of 15 who had previously received at least 3 doses of OPV during the last poliovirus outbreak in Israel which occurred in 1988 [38]. The WHO recommends investigating the cause of all cases of acute flaccid paralysis (AFP) independently of age, but only uses the rate of those under 15 years old for monitoring quality of AFP surveillance. Thus, in practice, identification of virus in stools of individuals with AFP>15 years old is inadequately investigated. We therefore recommend that all cases of adult AFP be fully evaluated in highly immunized populations.

cVDPV poliomyelitis outbreaks have occurred in communities where the expected survival times for immunodeficient individuals was short and the number of unprotected individuals high[39]. The relative risk for host-to-host transmission of disease in a population with high immunity is low but could occur within pockets of non-immunized individuals or between individuals with waned immunity. Determining the time of initial exposure to vaccine from a cluster of related aVDPVs may allow assessment of the relative risk of introduction of the aVDPV into external communities not as well covered by vaccination. For example, 1988 is a pivotal year for determining risks for re-emergence of poliomyelitis based on age of the infected individual(s) assuming that the initial exposure and evolution of Group 1 aVDPVs occurred in Israel. The initial exposure to Sabin 2 for Group 1 isolates occurred between 1985 and 1993 depending on the method used to calculate this date. Many factors interfere with more accurate approximation of starting dates for evolution and preclude use of elaborate correction algorithms [28] (discussed in the supplement). In Israel, most individuals ≤40 years old were vaccinated with OPV in 1988 as part of containment of the last wild type poliomyelitis outbreak [38]. In 1988, evolution of VDPV may have commenced in anyone ≤40. However, after 1988, exposure would most likely have occurred in children <8 year olds. This cohort has now entered the age of Israeli youth who travel abroad under hygienically compromised conditions in tropical areas with under-immunized populations. The sudden appearance of highly diverged Group 2 aVDPVs in more than 1 geographical region within a very short time resembles the sudden appearance of wild type polio into Israel and the Gaza district [4]. Thus by analogy, the Group 2 aVDPV's more likely represent introduction(s) rather than local circulation. Isolation of highly diverged Group 2 aVDPVs in sewage from a trunk line mainly serving hotels along the Mediterranean in the Tel Aviv area one month after finding a related isolate in the Jerusalem might point to a tourist or tourists as the vector. If true, this justifies our concern about the potential for global spread of aVDPVs like those in Group 1 by youthful tourists visiting regions of the world with sub-optimal vaccine coverage.

In conclusion, aVPDVs potentially circulate or are excreted for extended durations even in very intensively OPV vaccinated populations with high documented immunities. Taken together with the inability to cure chronically infected individuals [40] plans for cessation of poliovirus vaccination after eradication of wildtype poliomyelitis must be carefully reassessed. In fact vaccination with IPV may have to be continued until all individuals infected with VDPV cease excreting virus. Finally supplementary environmental sampling is a powerful surveillance tool for detecting poliovirus activity before appearance of AFP disease caused by re-emerging revertant vaccine strains.

## Materials and Methods

### Reference poliovirus

Type 1, 2, and 3 inactivated poliovaccine [PV1/Mahoney/USA41, PV2/MEF/EGY42, and PV3/Saukett/USA] and OPV [PV1/LSc 2ab, PV2/P712 ch 2ab, and PV3/Leon 12 a1b] reference strains and a minimally diverged, highly neurovirulent Sabin-1 positive neurovirulence control strain S139C6 [29]were obtained from Radu Crainic, Institute Pasteur, Paris, France.

### The sewage systems

The study was performed on sewage collected prior to entry into a treatment plant of central Israel that processes 120 million cubic meters of raw sewage per year and from sewage from the Jerusalem area prior to treatment by 2 plants which process 35 million cubic meters of raw sewage per year. The central Israel system serves approximately 1.6 million residents of diverse socio-economic backgrounds including new immigrants, tourists, and documented and undocumented migrant workers. The portion of the Jerusalem area described here services 700,000 residents with a background similar to that of Tel Aviv area residents. Central Israeli communities serviced by the central Israel treatment plant in 1998 and 2005 and sampling sites are shown in Figure S1.

### Isolation of polioviruses from sewage samples

Composite sewage samples of one liter generated by combining aliquots obtained automatically over a 24 hr interval were harvested each month from most Israeli sites and treated as detailed in the supplement. Treated samples were applied onto L20B cells. Grab samples collected during 3 hours of peak use were harvested from the Jerusalem area and a few other sentinel sites. The cell monolayers were overlaid with 1% agar and the plaques that formed were isolated for further processing as detailed in a supplement.

The majority (>98%) of the polioviruses isolated from sewage surveillance since 1989 have been OPVs excreted by recently immunized individuals or contacts. Imported wild poliovirus isolates were found sporadically in the Gaza District between 1990–2002 [4], [23] , L. M. Shulman et al, unpublished.], and in Israel in 1995 [4], [23] . This report focuses on the 9 highly diverged type 2 VDPV that were obtained from central Israel between 1998 and 2005 [8] [26]. For convenience all VDPV isolate names have been shortened to “SD” for Sabin-derived, a two digit number indicating the year of isolation, and another digit indicating the order of isolation with in a year if there were more than one isolate (i.e. pv2-5104-1_isr99 being the third isolate in 1999 has been renamed SD-99-3).

Intratypic differentiation, the process for determining whether a particular poliovirus isolate is vaccine-like (Sabin-like) or non-vaccine-like (non-Sabin-like) is described in the supplement.

### Sequence analyses

Sequence analyses were performed as described in detail in the supplement. The location of genomic fragments that were amplified by RT-PCR and sequenced for phylogenetic analysis is shown in Fig. 1.

### Phylogenetic analysis

Maximum likelihood phylogenetic trees representing different genomic regions were prepared using the Dnaml application of PHYLIP (J Felsenstein, Depart of Genetics, University of Washington, http://evolution.genetics.washington.edu/phylip/getme.html) . Prior to analysis, data was bootstrapped100 times and trees generated by randomizing sequence order 3x per bootstrap. Bootstrapping is a statistical method of resampling sequence data to estimate error and evaluate the reliability of a phylogenetic tree.

A neighbor joining phylogenetic tree was constructed using the ClustalX [41] program with sequence data bootstrapped 1000 times and visualized using njplot (M. Gouy, Laboratoire de Biometrie et Biologie Evolutive, U. Lyon, CNRS, France, URL http://pbiol.univlyon1.fr/software/njplot.html).

Since the sequences of the VDPVs are Sabin-derived, it was possible to root the trees to the corresponding Sabin sequences.

The frequencies of identical substitutions relative to OPV references and identity analysis of regions containing recombinations were determined using the NICER program (L. Shulman L and J. Prilusky, Israel, URL http://bioportal.weizmann.ac.il/nicer/). Similarity plots in the supplement were obtained by the SimPlot program [42].

### Geometric Mean Titers (GMT) to type 2 polioviruses

Sera from 8 anonymous Israeli cohorts 15 months to 50 years of age were obtained from district health offices as part of the Health Ministry annual sero-survey program or from the serum bank of the Israel Center for Disease Control (ICDC). The number of samples in each cohort, the year the samples were obtained and the vaccine status for the members of each cohort are summarized in Table 2. Titers of antibodies in serum that neutralized 100 TCID_50_ of Sabin 2, MEF, SD-98 and isolates SD-99-1, and SD-04 were determined by a micro-neutralization assay [30] and normalized to a pool of 20 positive sera in each test run. Neutralizing antibody titers against MEF, SD-98, SD-06-1 and SD-06-2 were determined for additional cohorts. Titers below or above starting and ending dilutions were assigned values of 1/2 the lowest or twice the highest dilution respectively, for GMT calculation. Significance of titer differences between different type 2 strains within each cohort and for the same strain between different cohorts, respectively was calculated using the Wilcoxon signed rank tests for paired data and unpaired data.

### Neurovirulence testing in transgenic mice

The magnitude of neurovirulence was determined in PVR-Tg21 transgenic mice [8], [29] (provided by A. Nomoto and T. Nomura). Five male and five female mice per poliovirus isolate were challenged i.p. with 0.33−1×10^8^ PFU and monitored daily for paresis, paralysis, or death over a 14 day interval. The Mean Health Time, defined as the mean number of days before appearance of clinical symptoms, was assayed.

### Nucleotide sequence accession numbers

aVDPV sequences have EMBL access numbers, AJ288062, AM040035 -39, AM056049-50, AM158275-6, and AM292219-21, while those for Sabin 1 and Sabin 2 are V01150 and X00595, respectively. The EMBL alignment access number is DS 63108.

## Supporting Information

Supplement S1 Electronic Supplement to this paper.
(7.76 MB DOC)

Figure S1 Communities serviced by the sewage system in central Israel. he area within the rectangle on the map of Israel (insert) which represents the greater Tel Aviv area and major surrounding areas in central Israel has been enlarged to show the areas serviced by the sewage system in 1998 (grey) and those added by 2004 (hashed lines). The Primary Surveillance Site (Site #1), located immediately before entry of sewage into the main treatment plant, services approximately 1.6 million individuals and has been sampled monthly since 1988. Five Secondary Surveillance Sites at the mouth of major trunk lines located upstream from the primary site (indicated by the smaller numbered circles) were added to try and localize the source or sources of the VDPV. They also increase the chances for isolating VDPVs by decreasing the distance from the source (i.e., decreased physical factors responsible for loss of detection and decrease the amount by which the excreted virus is diluted by the sewage). Secondary Sites #1-A through 1-E serve 800,000, 117,000, 238,600, 221,000, and 100,000 individuals, respectively. Tertiary Site #1-A-1 serves 50,000 residents along the seashore. Hotels are the major source of the sewage for this site. Highly-diverged type 2 VDPV isolates SD-98, SD-99-1, SD-99-2, SD-99-3, SD-99-4 and SD-04 were isolated from Primary Site #1. SD-05-1, SD-05-2 and SD-05-3 were isolated from Secondary Site #1-A. SD-06-1 was isolated form Primary Site #2. SD-06-2 and SD-06-3 were isolated from Tertiary Site #1-A-1.
(0.05 MB PDF)

Figure S2 A Similarity Plot for the recombination site in the 3D polymerase genes of aVDPVs.The 3D polymerase gene sequences (includes all of regions R5 and R6 and the linker in between ; Manuscript Fig. 1) of Sabin-1, Sabin-2 and 7 VDPV were aligned using ClustalX. A plot of nucleotide similarity between the 3D polymerase gene of the first aVDPV to be isolated, SD-98, and the subsequent 6 aVDPVs and Sabin 1 and Sabin 2 strains was generated by the SimPlot program using a sliding window of 300 bp in steps of 30 nt with JC correction model for nucleotide substitution. The first nucleotide in the alignment corresponds to nt 5940 of Sabin 2 (Acc: X00595). Dark black trace  =  Sabin 1, light grey trace  =  Sabin 2, and the VDPV are traces with broken lines. The crossover point in the SimPlot between similarity to Sabin 2 and similarity to Sabin 1 corresponds to positions 18 and 19 in the IdentityPlot shown in Fig. 2 of the manuscript.
(0.04 MB PDF)

Table S1 Description of the type 2 VDPV isolated from sewage between 1998 and 2006.
(0.03 MB DOC)

Table S2 Pair-wise nucleotide homology among aVDPVs and Sabin 2.
(0.18 MB DOC)
