# Supplementary material for: Neurovirulent Vaccine-Derived Polioviruses in Sewage from Highly Immune Populations
Source: PLoS One. 2006 Dec 20;1(1):e69. doi: 10.1371/journal.pone.0000069 (PMC1762338; doi:10.1371/journal.pone.0000069)
Supplement: Supplement S1 — Electronic Supplement to this paper. (7.76 MB DOC) [file pone.0000069.s001.doc]

Electronic Supplement to: Neurovirulent Vaccine-derived Polioviruses in Sewage from a Highly Immune Populations


Lester M. Shulman, Yossi Manor1, Danit Sofer1 Rachel Handsher1, Tiberio Swartz2, Francis Delpeyroux 3, and Ella Mendelson1,4.

The sewage systems. This study was performed on virus isolated from 2 separate sewage systems indicated by the numbers 1 and 2 on the map of Israel Figure. S1. The first system is located in the center of Israel on the coastal plain in the Tel Aviv region. The second is located in the hills in and around Jerusalem. 
The first area includes Tel Aviv and various municipalities surrounding Tel Aviv. The municipal borders of these communities are shown in the enlarged portion of the map equivalent to the boxed area on the Map of Israel in Figure S1. Primary Site # 1 is located immediately before the main sewage treatment plant in central Israel. This plant processes 120 million cubic meters of raw sewage per year. The system serves approximately 1.6 million residents of diverse socio-economic backgrounds including new immigrants, tourists, and documented and undocumented migrant workers. A number of trunk lines serving some adjacent municipalities were connected to the system between 1988 and 2005. This is also indicated on the map by hatched areas. Secondary Sites #1-A through #1-E were added to the monthly monitoring program in 2004 to increase the chances of detection of aVDPVs and as part of an initial effort to pinpoint the location of the excrtetor(s). The secondary sites are located at the mouths of major trunk lines that eventually feed into primary Site #1. Tertiary Sites 1-A-1 to 1-A-3 further subdividing the sewage system in branch 1A were added in 2006. 
The second system included the major portion of the Jerusalem area population. It services 700,000 of the total number of residents in the Jerusalem area. These residents have a background similar to those of Tel Aviv area residents. The sample containing aVDPV was a pool of sewage from 2 of the 3 major trunk lines in the Jerusalem area. Sewage from these lines is treated in 2 plants which together process 35 million cubic meters of raw sewage per year.  
An in-line composite sampler automatically collects an aliquot of sewage every hour for 24 hours at Primary Site #1. Sewage at Secondary Sites #1-A to #1-E, and Tertiary Sites #1-A-1 to #1-A-3 is collected over 24 yours as above using a portable automatic sampler. Sewage in Primary Site #2 is by grab sampling over 3 hours of peak usage.

Detailed procedure for isolation of polioviruses from sewage samples
Polyethylene glycol 6000 (Sigma or Merck) 80 grams and 17.5 grams of NaCl were added to 1 L of 24 hour composite sewage samples, at pH 7.2, by magnetic stirring. After storage overnight at 4 C, the samples were centrifuged  (10,000 x g, 1 hour) to obtain the insoluble fraction containing the precipitated poliovirus. This pellet was suspended in 15 ml PBS containing 0.1% Tween 80 and 15 ml chloroform to solubilize virus. The suspension was centrifuged at 3000 rpm for 15 minutes to collect the supernatant. The pellet was resuspended in 5 ml of 3% beef extract, pH 7.2 and centrifuged at 3000 rpm for 15 minutes. The supernatants from the two centrifugations were pooled to form the viral suspension.
Processed sewage was first inoculated onto transgenic murine L20B cells that express the human poliovirus receptor [1] to ensure that almost all viruses that grew are polioviruses. L20B cells, provided by the WHO, were challenged with the viral suspension and overlaid with 1% agar for individual plaque isolation. All plaques were re-grown on HEp2C at 40° C to select for non-vaccine viruses [2]. The WHO has recommended that both molecular and antigenic tests be used for intratypic differentiation. Isolates that grew within 5 days were characterized by typic and intra-typic neutralization, dot blot hybridization [3], and sequencing as previously described[4]. Initial genomic sequence comparison start with partial or complete viral capsid protein 1 (VP1) sequences. In dotblot hybridization, 4 aliquots of RNA from individual isolates are immobilized on a nylon filter and hybridized separately with specific RNA probes, one for each serotype and a 4th probe that identifies all enteroviruses. Hybridization conditions have been adjusted so that hybridization only occurs between spotted RNA from poliovaccine that has diverged by <1% and one of the three serotype-specific vaccine probes plus the enteroviral generic probe. When only generic probe binds, the virus is VDPV (more than 1% difference), a wild poliovirus (more than 15 % difference), or very rarely a non-polio enterovirus.  Sequence analysis differentiates between these three possibilities. The antigenic test employed in the Central Virology laboratory is a neutralization test using monoclonal or polyclonal antibodies that specifically neutralize vaccine strains but not non-vaccine strains. An additional feature that helps distinguish vaccine from non-vaccine polioviruses is that Sabin strains were “cold adapted” i.e., do not grow well at 40° C, whereas most non-Sabin VDPV and wild type strains do[5].
Uniform quality of processed sewage samples was verified based on WHO recommendations[6] that good environmental surveillance find enterovirus in  > 30 % of sewage samples. Starting in 2002 we verified the quality of all sewage samples by quantification of enterovirus plaques on BGM cells overlaid with 1% agar. Sensitivity of cell lines to viral infection was maintained and documented as required by the WHO[7].

aVDPV isolates. Table S1 provides a short descriptive summary of the 9 aVDPVs isolated from sewage of central Israel between 1998 and 2006. 

Sequence analyses. Molecular intratypic differentiation is based on comparison of a capsid protein 1 (VP1) sequence of an isolate with that of its corresponding Sabin vaccine serotype. If this comparison indicates that the isolate is a non-Sabin strain (>1% divergence of sequence), then three additional areas of the genome are sequenced. These regions represent non-coding regulatory regions (the 5' untranslated region at the beginning of the genome, 5'UTR), regions encoding other structural genes (the P1 region that encodes all capsid proteins including VP1) and regions encoding non-structural proteins (the viral encoded RNA polymerase located at the 3' end to the genome) since they might be subjected to different selective pressures during evolution and because recombination events are more likely to occur in non-coding and nonstructural genomic regions. The location of genomic fragments that were amplified by RT-PCR and sequenced for phylogenetic analysis is shown on the schematic diagram of the 7400 nt single stranded RNA genome of poliovirus manuscript Fig. 1. The SD-98, SD-99-1, 99-2, 99-3, and 99-4 were completely sequenced. 
Both strands of RT-PCR products (contact corresponding author for primer sequences) of extracted viral RNA [8], were sequenced using ABI PRISM Dye Deoxy Terminator Cycle Sequencing Kits (Applied Biosystems, Foster City, CA) in an Applied Biosystems Model 373 DNA Automatic Sequencing System. Nucleotide sequences were compared to Sabin sequences using the Sequencher program (Gencodes Corporation, Anne Arbor, MI, USA). The result of pairwise comparision of capsid protein VP1 for the P1 region encoding all capsid proteins is presented in Table S2. The amino acid sequences of neutralizing antigenic sites[8,9] were deduced using the DNA Strider program [Marck C. 1992. DNA Strider program, Service de Biochem, Institut de Recherche Fondamental, CEA, Saclay, France]. 
Circulating polioviruses also frequently recombine with genomes of other polio and non-polio enteroviruses in primary or subsequent hosts [10-13]. Recombination junctions generally clustered in particular subgenomic regions that were dependent on the serotype of the isolate and/or on the associations of genomic segments in recombinants[14].  However, since these preferential recombination sites are not very precise hot spots, it is broadly accepted that shared recombinations imply common ancestry. The recombination within the RNA polymerase gene demonstrated by the Identity Plot using the NICER program (Manuscript, upper panel of Fig. 2) can also be found by Similarity analysis using programs such as SimPlot [15]. An example of a SimPlot for the RNA polymerase is shown in Fig. S2. The crossover in similarity from Sabin 2 to Sabin 1 corresponds to the crossover in the IdentityPlot (Manuscript Fig. 2). 
Substitutions accumulate at a regular enough frequency in progeny of RNA viruses so that the number of changes can be used as a rough clock to estimate when the progenitor vaccine isolate was administered (see discussion below). The rate and pattern of substitutions is based on unique characteristics of the RNA primed RNA polymerases of these viruses. Like their wild counterparts, vaccines also accumulate substitutions at high rates. As a first estimation it is assumed that rates of substitutions are similar for wild viruses and live vaccines[9]. Essentially identical sequences are presented each time the vaccine is administered.  For poliovirus, OPV has been in use for at least 50 years (www.cdc.gov/niP/webutil/about/annual-rpts/ar2005/special-report-2005.pdf). This means that progeny of individual vaccine isolates administered at different times or to different individuals will accumulate near identical numbers of substitutions within a given time post vaccination. Relative homology indicates how closely the vaccine-derived isolates are to the vaccine strain. However, it is the pattern (number and distribution) of identical substitutions relative to vaccine progenitor and the appearance and presence of unique genomic recombination markers that indicate whether 2 or more vaccine derived isolates evolved from the same vaccine isolate. The NICER program identifies the distribution pattern and frequencies of identical substitutions in query sequences compared with one or 2 reference strain sequences (http://bip.weizmann.ac.il/nicerb/main). 
 
Evaluation of estimations of the time of the initiating exposure. The initial exposure to Sabin 2 occurred between 1985 and 1993 depending on the method used to calculate this date. Many factors interfere with more accurate approximation of starting dates for evolution and preclude use of elaborate correction algorithms [9] . These include the following: 1. Unknown source(s) excrete the aVDPV so that the appropriateness of correction factors can not be corroborated against known vaccination dates as for iVDPV[9]. 2. It is unclear when creation of a chimeric type2:type1 polymerase by recombination occurred other than that it occurred prior to isolation of the 1st VDPV in 1998, and whether its creation effected the linear rate of misincorporation. 3. As the time interval between isolations increases, sites begin to be re-substituted resulting in underestimation of the number of substitutions in proportion to circulation times. 4. The number of isolates is small and not uniformly distributed over the interval of observation, increasing bias due to clusters of similar sequences. 5. The isolates are single plaque-isolates from the environment with all of the uncertainties involved in sampling[16] and they may not represent the mainline sequence of the quasispecies as is the case when sampling stools from an immunodeficient individual[9], And 6. The high number of identical substitutions versus the high inter-lineage divergence with possible complication from intratypic recombination makes it difficult to determine the time the lineages started to diverge.
Identification of individual excretors of aVDPV is important since it may provide sequence data and immunization history potentially valuable for validating the calculated dates of initial exposure. All 23 chronically infected poliovirus excretors identified worldwide to date have been individuals with primary B-cell immune deficiency disorders[17]. No additional chronic excretors were found in a global search of 300 immune deficiency patients[18]. Similarly, no chronically infected individuals have been identified by enteroviral surveillance in Israel between 1998 and 2005, especially cases with neurological involvement and acute flaccid paralysis. This finding is consistent with absence of a widespread circulation of neurovirulent aVDPV.  Gradual tracing the site of entry of VDPVs by successively adding new sample sites upstream in sewer systems is laborious and time consuming. Furthermore since detection is intermittent, it may be unlikely to succeed. 


1. Pipkin PA, Wood DJ, Racaniello VR, Minor PD (1993) Characterisation of L cells expressing the human poliovirus receptor for the specific detection of polioviruses in vitro. J Virol Methods 41: 333-340.
2. Manor Y, Handsher R, Halmut T, Neuman M, Abramovitz B, et al. (1999) A double-selective tissue culture system for isolation of wild-type poliovirus from sewage applied in a long-term environmental surveillance. Appl Environ Microbiol 65: 1794-1797.
3. De L, Nottay B, Yang CF, Holloway BP, Pallansch M, et al. (1995) Identification of vaccine-related polioviruses by hybridization with specific RNA probes. J Clin Microbiol 33: 562-571.
4. Shulman LM, Manor Y, Handsher R, Delpeyroux F, Halmut T, et al. Characterization of a highly evolved Sabin 2 polio strains isolated from sewage; 2001 2001; Bersheva, Israel. ISM News. pp. 34.
5. Nakano JH, Hatch MH, Thieme ML, Nottay B (1978) Parameters for differentiating vaccine-derived and wild poliovirus strains. Prog Med Virol 24: 178-206.
6. WHO t (2003) Guidelines for environmental surveillance of poliovirus  circulation. WHO, Dept of Vaccines and Biologicals.
7. WHO T (2005) Experience with cell sensitivity LQC is reviewed
. Polio Lab Network Quarterly Update
 X: 1-3.
8. Shulman LM, Manor Y, Handsher R, Delpeyroux F, McDonough MJ, et al. (2000) Molecular and antigenic characterization of a highly evolved derivative of the type 2 oral poliovaccine strain isolated from sewage in Israel. J Clin Microbiol 38: 3729-3734.
9. Yang CF, Chen HY, Jorba J, Sun HC, Yang SJ, et al. (2005) Intratypic recombination among lineages of type 1 vaccine-derived poliovirus emerging during chronic infection of an immunodeficient patient. J Virol 79: 12623-12634.
10. Shulman LM, Manor Y, Alfandari J, Kilpatrick DR, Halmut T, et al. (2004) Rapid evolution by recombination of OPV strains in immunized children in an urban Bedouin community. Program and Abstracts of the Annual Meeting of The Israeli Society of Microbiology, Haifa, Israel Feb 9-10, 2004
: 29.
11. Wimmer E, Hellen CU, Cao X (1993) Genetics of poliovirus. Annu Rev Genet 27: 353-436.
12. Agol VI (1997) Recombination and Other Genomic Rearrangements in Picornaviruses
. Seminars in Virology   8: 77-78.
13. Guillot S, Caro V, Cuervo N, Korotkova E, Combiescu M, et al. (2000) Natural genetic exchanges between vaccine and wild poliovirus strains in humans. J Virol 74: 8434-8443.
14. Cuervo NS, Guillot S, Romanenkova N, Combiescu M, Aubert-Combiescu A, et al. (2001) Genomic features of intertypic recombinant sabin poliovirus strains excreted by primary vaccinees. J Virol 75: 5740-5751.
15. Lole KS, Bollinger RC, Paranjape RS, Gadkari D, Kulkarni SS, et al. (1999) Full-length human immunodeficiency virus type 1 genomes from subtype C-infected seroconverters in India, with evidence of intersubtype recombination. J Virol 73: 152-160.
16. Ranta J, Hovi T, Arjas E (2001) Poliovirus surveillance by examining sewage water specimens: studies on detection probability using simulation models. Risk Anal 21: 1087-1096.
17. Kew OM, Sutter RW, de Gourville EM, Dowdle WR, Pallansch MA (2005) Vaccine-Derived Polioviruses And The Endgame Strategy For Global Polio Eradication (*). Annu Rev Microbiol 59: 587-635.
18. Halsey NA, Pinto J, Espinosa-Rosales F, Faure-Fontenla MA, da Silva E, et al. (2004) Search for poliovirus carriers among people with primary immune deficiency diseases in the United States, Mexico, Brazil, and the United Kingdom. Bull World Health Organ 82: 3-8.


Figure and Table Legends

Figure S1. Communities serviced by the sewage system in central Israel. 

The area within the rectangle on the map of Israel (insert) which represents the greater Tel Aviv area and major surrounding areas in central Israel has been enlarged to show the areas serviced by the sewage system in 1998 (grey) and those added by 2004 (hashed lines). The Primary Surveillance Site (Site #1), located immediately before entry of sewage into the main treatment plant, services approximately 1.6 million individuals and has been sampled monthly since 1988.  Five Secondary Surveillance Sites at the mouth of major trunk lines located upstream from the primary site (indicated by the smaller numbered circles) were added to try and localize the source or sources of the VDPV. They also increase the chances for isolating VDPVs by decreasing the distance from the source (i.e., decreased physical factors responsible for loss of detection and decrease the amount by which the excreted virus is diluted by the sewage). Secondary Sites #1-A through 1-E serve 800,000, 117,000, 238,600, 221,000, and 100,000 individuals, respectively. Tertiary Site #1-A-1 serves 50,000 residents along the seashore. Hotels are the major source of the sewage for this site. 
Highly-diverged type 2 VDPV isolates SD-98, SD-99-1, SD-99-2, SD-99-3, SD-99-4 and SD-04 were isolated from Primary Site #1.  SD-05-1, SD-05-2 and SD-05-3 were isolated from Secondary Site #1-A.  SD-06-1 was isolated form Primary Site #2. SD-06-2 and SD-06-3 were isolated from Tertiary Site #1-A-1.


Figure S2. A Similarity Plot for the recombination site in the 3D polymerase genes of aVDPVs.

The 3D polymerase gene sequences (includes all of regions R5 and R6 and the linker in between ; Manuscript Fig. 1) of Sabin-1, Sabin-2 and 7 VDPV were aligned using ClustalX. A plot of nucleotide similarity between the 3D polymerase gene of the first aVDPV to be isolated, SD-98, and the subsequent 6 aVDPVs and Sabin 1 and Sabin 2 strains was generated by the SimPlot program using a sliding window of 300 bp in steps of 30 nt with JC correction model for nucleotide substitution. The first nucleotide in the alignment corresponds to nt 5940 of Sabin 2 (Acc: X00595). Dark black trace = Sabin 1, light grey trace = Sabin 2, and the VDPV are traces with broken lines. The crossover point in the SimPlot between similarity to Sabin 2 and similarity to Sabin 1 corresponds to positions 18 and 19 in the IdentityPlot shown in Fig. 2 of the manuscript.


Table S1. Description of the type 2 VDPV isolated from sewage between 1998 and 2006.

a.	Refers to Primary, Secondary or Tertiary Sites in the sewage system as shown in SFigure 1.

Table S2. Pair-wise nucleotide homology among aVDPVs and Sabin 2. 

Numbers indicate the % differences for each pair-wise comparison of aVDPVs among themselves and with Sabin 2 for either all capsid proteins (P1) or capsid protein VP1. P1 and VP1 correspond to regions R3 and R4, respectively, in Manuscript Fig. 1. 


Table 1:  Description of the type 2 VDPV isolated from sewage between 1998 and 2006. 

Isolation Date	Isolate full name	Isolate short name	Sampling sitea	Accession number	
May 05, 1998	PV2/4568-1/ISR98	SD-98	Site #1	AJ288062 AM040035	
Sep 06, 1999	PV2/5021-1/ISR99	SD-99-1	Site #1	AM040036	
Nov 03, 1999	PV2/5074-18/ISR99	SD-99-2	Site #1	AM040037	
Dec 06, 1999	PV2/5104-1/ISR99	SD-99-3	Site #1	AM040038	
Dec 15, 1999	PV2/5116-9/ISR99	SD-99-4	Site #1	AM040039	
Apr 22, 2004	PV2/6056-3/ISR04	SD-04	Site #1	AM056049	
Apr 13, 2005	PV2/6316-1/ISR05	SD-05-1	Site # 1-A	AM056050	
Jul 20, 2005	PV2/6389-1/ISR05	SD-05-2	Site # 1-A	AM158275	
Jul 20, 2005	PV2/6389-6/ISR05	SD-05-3	Site # 1-A	AM158276	
Feb 02, 2006	PV2/6526-1/ISR06	SD-06-1	Site #2	AM292219	
Mar 14, 2006	PV2/6560-1/ISR06	SD-06-2	Site # 1-A-1	AM292220	
Mar 14, 2006	PV2/6560-2/ISR06	SD-06-3	Site # 1-A-1	AM292221	
