# Supplementary figures and images for: Neurovirulent Vaccine-Derived Polioviruses in Sewage from Highly Immune Populations
Source: PLoS One. 2006 Dec 20;1(1):e69. doi: 10.1371/journal.pone.0000069 (PMC1762338; doi:10.1371/journal.pone.0000069)

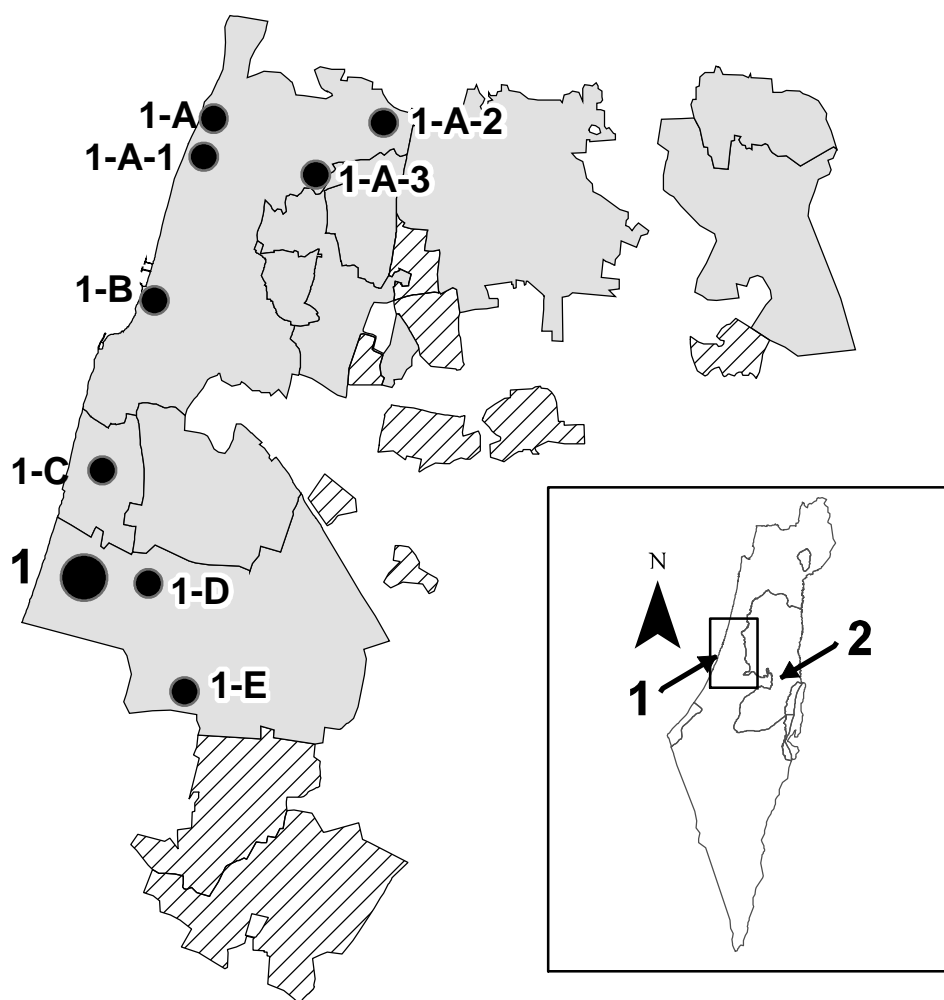

## Legend

1998

Primary Site

Added by 2004

Secondary Sites

0 2.5 5 10 15 20 Kilometers

Supplement: Figure S1 — Communities serviced by the sewage system in central Israel. he area within the rectangle on the map of Israel (insert) which represents the greater Tel Aviv area and major surrounding areas in central Israel has been enlarged to show the areas serviced by the sewage system in 1998 (grey) and those added by 2004 (hashed lines). The Primary Surveillance Site (Site #1), located immediately before entry of sewage into the main treatment plant, services approximately 1.6 million individuals and has been sampled monthly since 1988. Five Secondary Surveillance Sites at the mouth of major trunk lines located upstream from the primary site (indicated by the smaller numbered circles) were added to try and localize the source or sources of the VDPV. They also increase the chances for isolating VDPVs by decreasing the distance from the source (i.e., decreased physical factors responsible for loss of detection and decrease the amount by which the excreted virus is diluted by the sewage). Secondary Sites #1-A through 1-E serve 800,000, 117,000, 238,600, 221,000, and 100,000 individuals, respectively. Tertiary Site #1-A-1 serves 50,000 residents along the seashore. Hotels are the major source of the sewage for this site. Highly-diverged type 2 VDPV isolates SD-98, SD-99-1, SD-99-2, SD-99-3, SD-99-4 and SD-04 were isolated from Primary Site #1. SD-05-1, SD-05-2 and SD-05-3 were isolated from Secondary Site #1-A. SD-06-1 was isolated form Primary Site #2. SD-06-2 and SD-06-3 were isolated from Tertiary Site #1-A-1. (0.05 MB PDF) [file pone.0000069.s002.pdf]

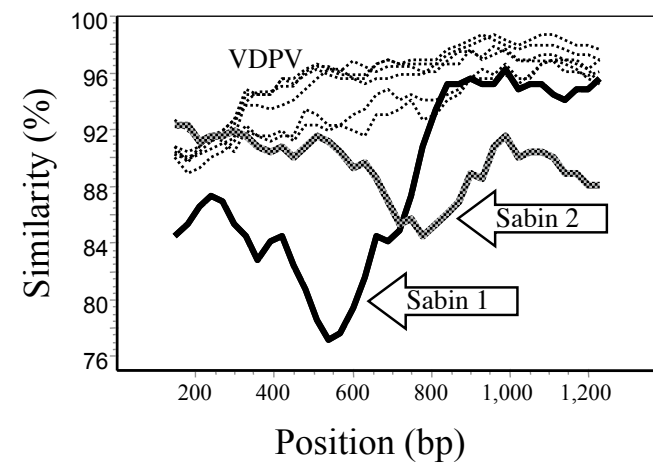

Supplement: Figure S2 — A Similarity Plot for the recombination site in the 3D polymerase genes of aVDPVs.The 3D polymerase gene sequences (includes all of regions R5 and R6 and the linker in between ; Manuscript Fig. 1) of Sabin-1, Sabin-2 and 7 VDPV were aligned using ClustalX. A plot of nucleotide similarity between the 3D polymerase gene of the first aVDPV to be isolated, SD-98, and the subsequent 6 aVDPVs and Sabin 1 and Sabin 2 strains was generated by the SimPlot program using a sliding window of 300 bp in steps of 30 nt with JC correction model for nucleotide substitution. The first nucleotide in the alignment corresponds to nt 5940 of Sabin 2 (Acc: X00595). Dark black trace = Sabin 1, light grey trace = Sabin 2, and the VDPV are traces with broken lines. The crossover point in the SimPlot between similarity to Sabin 2 and similarity to Sabin 1 corresponds to positions 18 and 19 in the IdentityPlot shown in Fig. 2 of the manuscript. (0.04 MB PDF) [file pone.0000069.s003.pdf]
