# Supplementary material for: Neurovirulent Vaccine-Derived Polioviruses in Sewage from Highly Immune Populations
Source: PLoS One. 2006 Dec 20;1(1):e69. doi: 10.1371/journal.pone.0000069 (PMC1762338; doi:10.1371/journal.pone.0000069)
Supplement: Table S1 — Description of the type 2 VDPV isolated from sewage between 1998 and 2006. (0.03 MB DOC) [file pone.0000069.s004.doc]

**Table S1. Description of the type 2 VDPV isolated from sewage between 1998 and 2006.**

| Isolation Date | Isolate full name | Isolate short name | Sampling site**a** | Accession number |
| --- | --- | --- | --- | --- |
| May 05, 1998 | PV2/4568-1/ISR98 | SD-98 | Site #1 | AJ288062 AM040035 |
| Sep 06, 1999 | PV2/5021-1/ISR99 | SD-99-1 | Site #1 | AM040036 |
| Nov 03, 1999 | PV2/5074-18/ISR99 | SD-99-2 | Site #1 | AM040037 |
| Dec 06, 1999 | PV2/5104-1/ISR99 | SD-99-3 | Site #1 | AM040038 |
| Dec 15, 1999 | PV2/5116-9/ISR99 | SD-99-4 | Site #1 | AM040039 |
| Apr 22, 2004 | PV2/6056-3/ISR04 | SD-04 | Site #1 | AM056049 |
| Apr 13, 2005 | PV2/6316-1/ISR05 | SD-05-1 | Site # 1-A | AM056050 |
| Jul 20, 2005 | PV2/6389-1/ISR05 | SD-05-2 | Site # 1-A | AM158275 |
| Jul 20, 2005 | PV2/6389-6/ISR05 | SD-05-3 | Site # 1-A | AM158276 |
| Feb 02, 2006 | PV2/6526-1/ISR06 | SD-06-1 | Site #2 | AM292219 |
| Mar 14, 2006 | PV2/6560-1/ISR06 | SD-06-2 | Site # 1-A-1 | AM292220 |
| Mar 14, 2006 | PV2/6560-2/ISR06 | SD-06-3 | Site # 1-A-1 | AM292221 |

1. Refers to Primary, Secondary or Tertiary Sites in the sewage system as shown in Figure S1.
