# Supplementary material for: Neurovirulent Vaccine-Derived Polioviruses in Sewage from Highly Immune Populations
Source: PLoS One. 2006 Dec 20;1(1):e69. doi: 10.1371/journal.pone.0000069 (PMC1762338; doi:10.1371/journal.pone.0000069)

### **Table S2**. **Pair-wise nucleotide homology among aVDPVs and Sabin 2.**

### Numbers indicate the % differences for each pair-wise comparison of aVDPVs among themselves and with Sabin 2 for either all capsid proteins (P1) or capsid protein VP1. P1 and VP1 correspond to regions R3 and R4, respectively, in Manuscript Fig. 1.


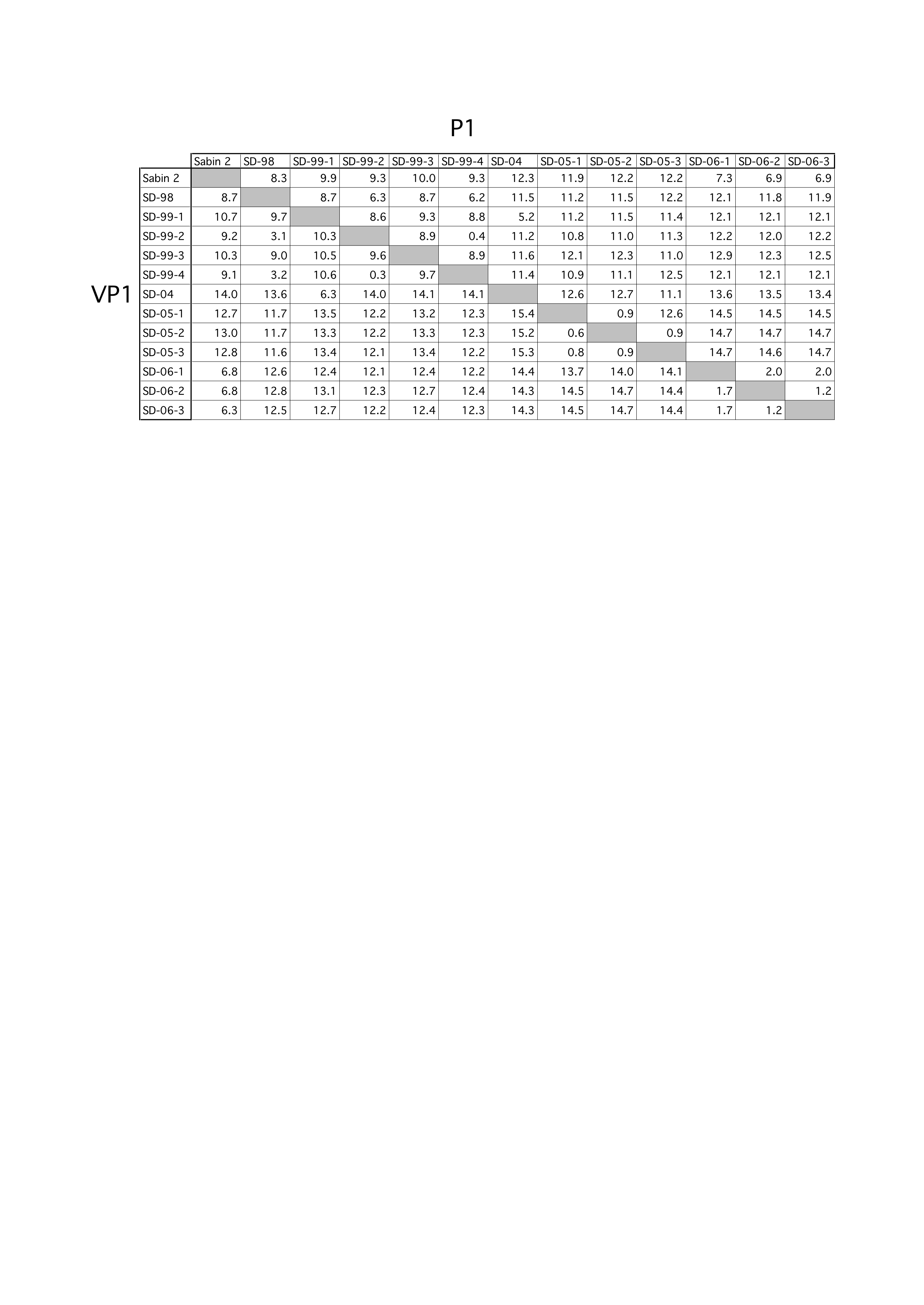

Supplement: Table S2 — Pair-wise nucleotide homology among aVDPVs and Sabin 2. (0.18 MB DOC) [file pone.0000069.s005.doc]
